# The role of human photosynthesis in predictive, preventive and personalized medicine

**DOI:** 10.1186/1878-5085-5-S1-A146

**Published:** 2014-02-11

**Authors:** Sergey Suchkov, Arturo Solís Herrera

**Affiliations:** 1 I.M. Sechenov First Moscow State Medical University (FMSMU), Moscow, Russia; 2 A.I.Konstantinov Moscow State Medical & Dental University, Moscow, Russia; 3 Human Photosynthesis Study Center, Lopez Velarde 108, Centro; Aguascalientes, Aguascalientes, CP 20000, México

## 

With human genome mapping, the omics revolution and the empowering sequencing technologies developed at the turn of the century, the new goals in medicine are to switch from population medicine to individualized therapies, not only to cure diseases but also to prevent them [1]. Diseases are complex processes that are caused by a combination of genetic and environmental factors. Uncovering the molecular pathways through which genetic factors affect a phenotype is always difficult, but in the case of complex diseases this is further complicated since genetic factors in affected individuals might be different.

Biomolecules in a living organism rarely act individually. Instead they work together in a cooperative way to provide specific functions. A variety of intermolecular interactions have been described including protein-protein interactions, protein-glycan interaction, protein-DNA interactions and RNA interactions that are essential to these cooperative activities.

It is widely accepted that the cellular system is modular, and a functional module is an entity, composed of many types of interacting molecules, whose function is separable, within certain limits, from those of other modules. There is no unique way to mathematically define functional modules [2], because metabolic pathways are often incomplete.

Networks of molecular interactions are trying to explain complex biological processes [3]. However in the scientific literature, metabolic and signaling pathways are often viewed separately, even both are composed of interactions involving proteins, glycan, and other chemical entities. It is necessary to combine data from major available resources to judge the functionality, complexity, completeness and manipulability of any given network overall, but the full integration of relevant information from scientific literature is still ongoing, complex and formidable task.

The discovery of the intrinsic property of melanin to dissociate and re-form the water molecule, a fact previously unknown, may be a factor that significantly facilitates the integration of current knowledge in relation to the sequence following biomolecules in an organism alive.

So far no reaction could consider some intracellular as number 1 or very first of all, but have identified the process very similar to the first reaction of plants implies a breakthrough in this regard. In plants, the first reaction of photosynthesis is through dissociation of the water molecule by chlorophyll, a reaction that is irreversible, since the oxygen is expelled into the atmosphere. The reaction is outlined as follows:

2H_2_O → 2H_2_ + O_2_

And it is considered the world's most important reaction since it is the beginning of the food chain. Therefore, a plant without water will not hatch, since the free chemical energy that is released with the breakdown of the molecule of water is essential to boost consequential reactions finally reaches the fusion of CO2 and H2O into glucose, a process that unable to replicate in the laboratory. We could say that every last leaf of the tree stems depends on and is governed by photosynthesis.

Our discovery of the intrinsic property of the Melanin molecule to dissociate and re-form the water molecule breaks the paradigm, humans also have the amazing ability to transform the photon energy into chemical energy free [4], and the reaction is outlined in the following form:

2H_2_O ↔ 2H_2_ + O_2_ + 4e^-^

Melanin is thousands of times more efficient to dissociate the water molecule of chlorophyll, it suffices to note that chlorophyll does so irreversible and can only use purple and red light, visible both, for our side, melanin absorbs the entire electromagnetic spectrum, apart from splitting water, also has the amazing ability to re-shape it, which is a unique example in nature. And just as in the plants until the last leaf of the tree stems, depends on and is governed entirely by photosynthesis, in humans is the same.

That is why we think we can assign as number 1, the dissociation reaction of water molecule also in humans. And in doing so, the cloud of unknowns surrounding the self-sustaining chemical system we call life, so substantially modified. Well now it has a beginning, and the intrinsic property of melanin to dissociate the water molecule has the appropriate requirements for considering the ab initio life [5].

So the real source of energy of the eukaryotic cell is water, so the sacred role of glucose as an energy source now breaks into a thousand pieces. We ended up saying that if the energy source was glucose, diabetic patients would fly.

The human body with four billion years of evolution is far beyond our ability to abstraction, but in origin the body is relatively simple: everything comes, everything is soaked, and everything is governed by photosynthesis, both in plants and in us.

If we want to maintain health, the answer is also simple: just keep the balance equation.

2H_2_O ↔ 2H_2_ + O_2_ + 4e^-^
